# Dural-Based Plasmacytoma Presenting As the Initial Sign of Multiple Myeloma

**DOI:** 10.7759/cureus.91986

**Published:** 2025-09-10

**Authors:** Khalid T Alghamdi, Alaa N Turkistani, Osama A Alkulli, Afnan Samman, Alaa Samkari, Ahmed I Lary

**Affiliations:** 1 Department of Neurosurgery, King Faisal Specialist Hospital and Research Centre, Riyadh, SAU; 2 Department of Neurosurgery, King Faisal Specialist Hospital and Research Centre, Jeddah, SAU; 3 College of Medicine, King Saud Bin Abdulaziz University for Health Sciences, Jeddah, SAU; 4 Department of Neurosurgery, King Abdulaziz Medical City, Jeddah, SAU; 5 Department of Pathology and Laboratory Medicine, King Abdulaziz Medical City, Ministry of National Guard Health Affairs, Jeddah, SAU; 6 Department of Neurosurgery, King Abdulaziz Medical City, Ministry of National Guard Health Affairs, Jeddah, SAU

**Keywords:** diagnosis of multiple myeloma, dural based plasmacytoma, mimicking meningioma, skull tumor, tumor skull extension

## Abstract

In multiple myeloma (MM), intracranial involvement is rare, particularly in cases of dural involvement. Cranial plasmacytoma can be seen in MM and may occasionally present as a solitary lesion. On MRI, dural plasmacytomas can mimic other extra-axial masses such as meningiomas, metastases, lymphomas, or sarcomas of the dura mater.

A 66-year-old woman presented with a right occipital swelling identified on an external MRI scan, which was accompanied by a headache. The MRI suggested MM or parieto-occipital intraosseous meningioma. The patient was advised to undergo a follow-up MRI, which she did not attend. Three months later, a head CT scan revealed that the mass had regressed spontaneously, and she remained asymptomatic. For that reason, she declined treatment. Five months later, the patient returned with left-sided hemiparesis. Follow-up imaging showed significant growth of the tumor. After tumor extraction, histopathological analysis with immunohistochemistry revealed a plasma cell neoplasm with lambda light chain.

Prior to making treatment decisions, it is crucial to conduct a thorough systemic evaluation and preoperative workup for MM when plasmacytoma is considered in the differential diagnosis.

## Introduction

Plasma cell tumors are considered rare malignancies in the head and neck region, accounting for approximately 0.04%. Plasma cell neoplasms most commonly occur in individuals during their sixth decade of life [1, 2]. Plasma cell malignancies can be classified into three types: (i) multiple myeloma (MM), (ii) solitary plasmacytoma of bone, and (iii) extramedullary plasmacytoma [3-7]. MM differs from the others by having systemic manifestations, and it accounts for 4 cases per 100,000 of the population [8]. It may arise de novo or progress from other types, such as solitary extramedullary plasmacytoma, with the progressive form associated with a low survival rate of 2 to 3 years [1, 9]. The novelty of this case lies in its unique presentation, in which the patient initially presented with a skull swelling that regressed in size and then began to progress again, with more intracranial extension causing neurological manifestations.

## Case presentation

**Figure 1:**
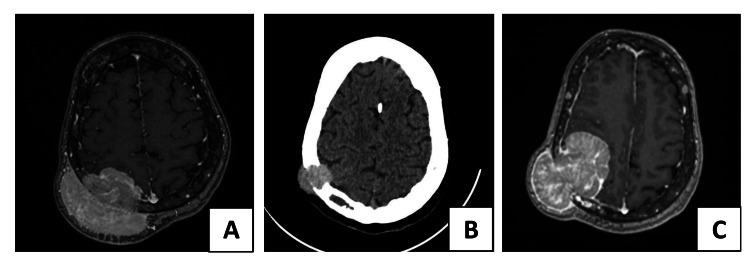
(A) Axial brain MRI at the time of presentation showing an exophytic, hyperintense mass extending intra- and extracranially. (B) Axial brain CT at the largest diameter of the tumor after spontaneous regression. (C) Brain MRI showing re-progression of the hyperintense tumor with greater intracranial invasion. By comparing the initial MRI (A) and the last MRI (C), there is clear regression of the extracranial component of the lesion; however, there is extensive progression of the intracranial component, which explains the patient’s clinical manifestations.

A 66-year-old woman, who was taking medication for hypertension, presented with a history of right occipital cranial enlargement and headaches that had persisted for eight months. MRI of the head was conducted, which suggested that the patient had a meningioma (Figure 1). The patient was offered surgery at that time; however, she and her family refused and chose observation. She was therefore advised to undergo a follow-up MRI and close follow-up after three months, with clear explanation of red flag symptoms including signs of raised intracranial pressure or development of new neurological deficits. However, the patient did not attend the MRI. She later presented to the clinic reporting progressive resolution of the lesion, which was consistent with physical examination and CT brain findings that revealed evidence of tumor regression. Consequently, the patient became more reluctant to undergo surgical treatment, and she was scheduled again for MRI follow-up (Figure 1). Five months later, the patient returned with complaints of general weakness on the left side, particularly in the upper limb (muscle power scale of 2/5), in addition to elements of left homonymous hemianopia. The patient was admitted, and repeat MRI demonstrated a substantial increase in tumor size. Based on the MRI findings, a differential diagnosis was proposed: histiocytosis, parieto-occipital intraosseous meningioma, or MM (Figure 1).

Surgical management 

**Figure 2:**
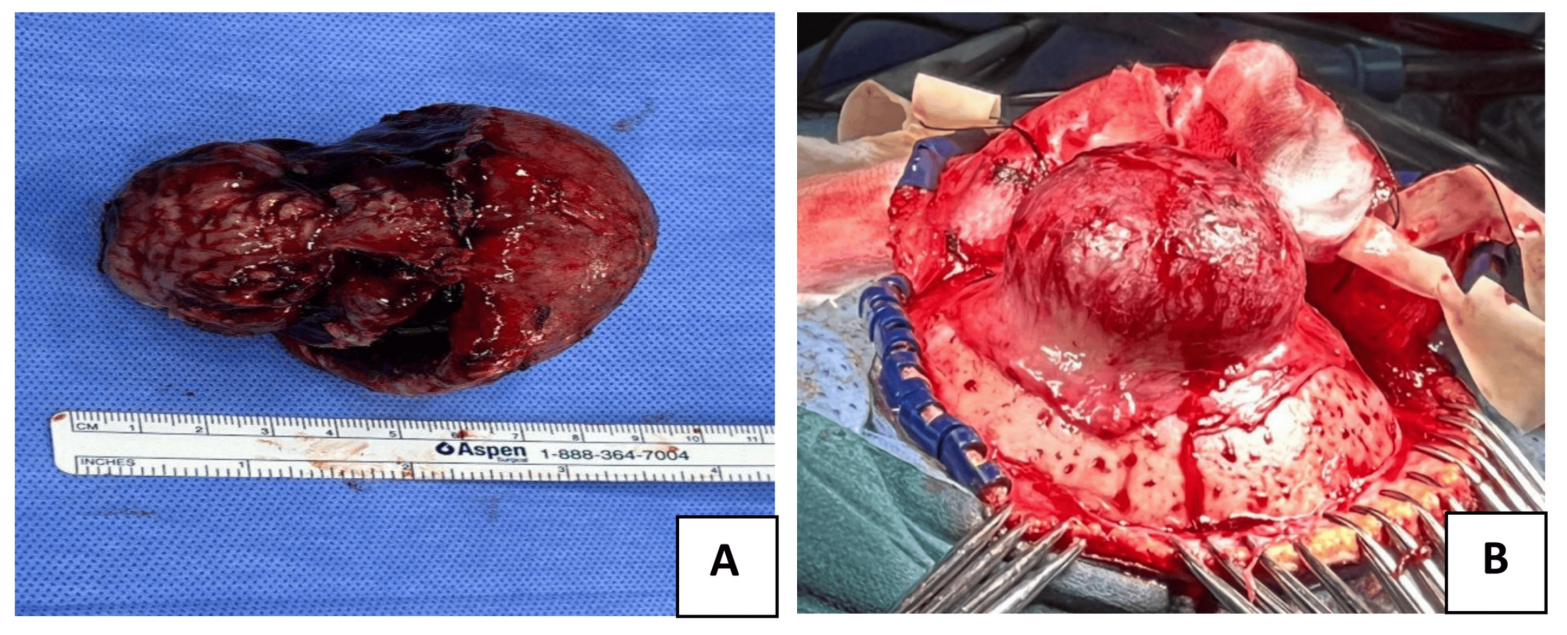
Images A and B show the intraoperative view and the resected specimen post-resection.

Gross total resection of the tumor was performed, including the surrounding unhealthy bone and dura mater. A highly vascular lesion was removed and sent for pathology (Figure 2). Postoperatively, the patient was stable with only mild weakness on the left side.

Histopathology 

**Figure 3:**
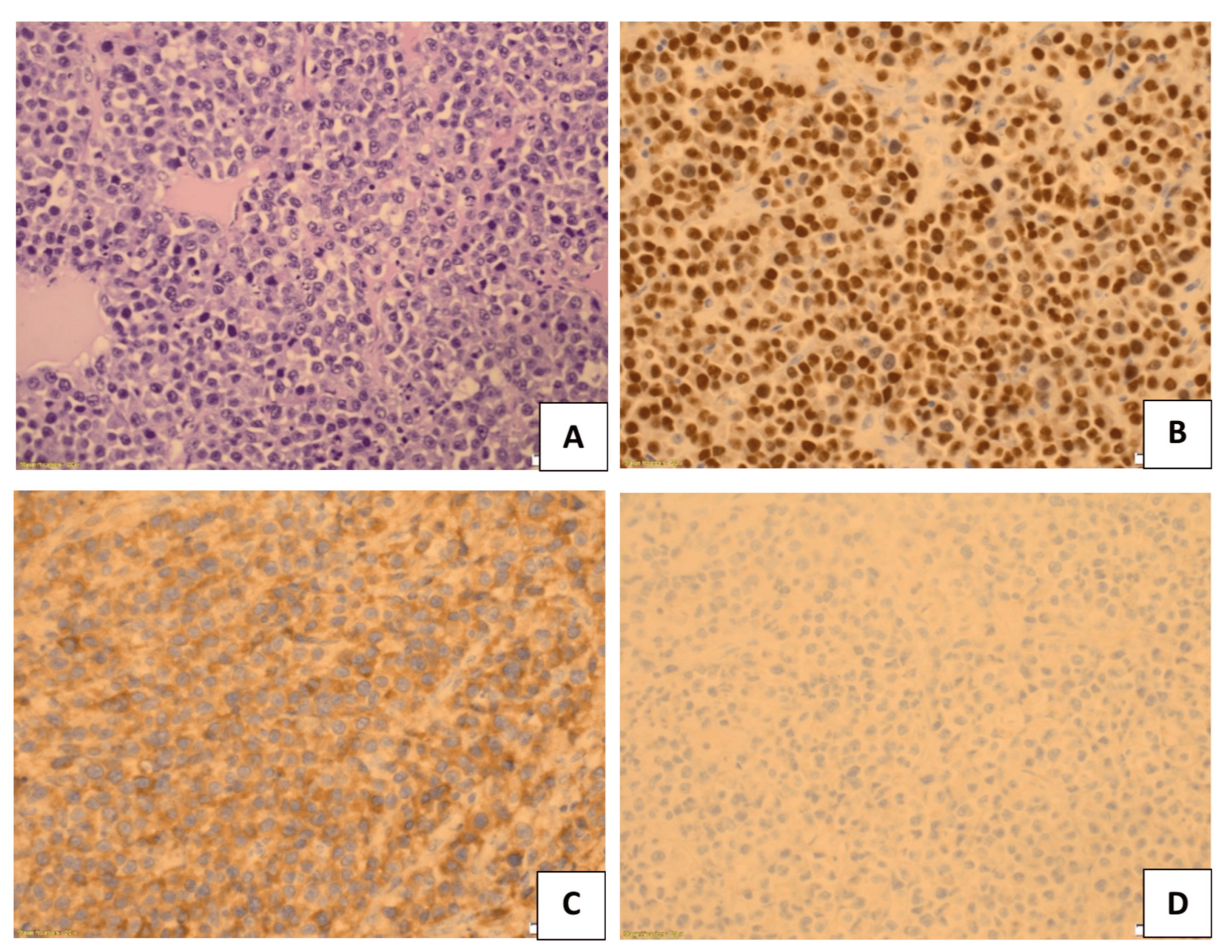
(A) H&E stain showing sheets of atypical plasma cells with apoptotic cells and mitotic figures. (B) Immunohistochemistry stain: MUM1 positive. (C) Immunohistochemistry stain: Lambda positive. (D) Immunohistochemistry stain: Kappa negative.

Microscopically, H&E staining revealed sheets of atypical plasma cells infiltrating the dura mater and the adjacent skull bone (Figure 3A). Apoptotic bodies and mitotic figures were observed. Immunohistochemical analysis demonstrated positive staining for MUM1, CD38, CD138, CD45, CD79a, and lambda light chains (Figures 3B-3D). Histopathological evaluation confirmed a plasma cell neoplasm with lambda light chain restriction on immunohistochemistry.

Follow-up

​After the lesion was excised, the patient was postoperatively stable, with only modest weakness on the left side (muscle power scale of 4/5). Her follow-up CT brain was unremarkable, showing no hemorrhage, infarction, or residual mass. However, bone marrow biopsy revealed evidence of MM. The patient was referred to medical oncology and subsequently underwent radiation therapy. Unfortunately, the patient’s MM was refractory to treatment, and she passed away after two years.

## Discussion

A dural-based plasmacytoma resembling a meningioma has been previously reported, often affecting both the leptomeninges and the skull at the same time [9-13]. In the literature, only a few cases have been described in which MM presented with intracranial plasmacytoma without any other evidence of disease. Solitary cranial plasmacytoma occurs rarely [14], as it usually presents with other manifestations of MM, as in our case [11, 15, 16]. The unique aspect of our case was the spontaneous regression followed by further progression within a few months. Such a presentation might give false reassurance to the patient and possibly to the physicians as well, which may delay treatment, as happened in our case.

Dural plasmacytomas may have distinctive radiologic features that can mimic meningiomas, lymphomas, or metastases. Therefore, histopathological examination is necessary for diagnosis. A definitive diagnosis requires excluding systemic MM and confirming the tumor’s monoclonal cell origin to differentiate it from inflammatory lesions [17]. Schwartz TH et al. investigated the relationship between cell adhesion molecules, plasmacytoma location, and the likelihood of progression to MM. They suggested that lesions at the cranial base are more predictive of subsequent MM, while extramedullary dural-based lesions are generally not linked to MM and should therefore be surgically removed [9]. However, in our case, the lesion was dural-based and ultimately proved to be the initial manifestation of MM.

The management of cranial plasmacytoma remains poorly defined due to its rarity in the literature. For solitary plasmacytomas, gross total resection followed by adjuvant radiotherapy is generally suggested as the recommended treatment. In contrast, therapeutic options for patients with plasmacytomas associated with MM remain limited [14, 18]. Many authors suggest starting treatment with a combination of chemotherapy and radiation as the first-line approach, given the high sensitivity of these lesions to this regimen. Surgery is typically reserved for refractory cases or when radiation is not feasible due to proximity to critical structures, such as the optic chiasm [9, 19]. In our case, gross total resection was performed prior to confirming the diagnosis of MM due to the severity of the patient’s neurological symptoms. However, generally speaking, preoperative evaluation for MM is crucial when plasmacytoma is suspected in the differential diagnosis.

## Conclusions

Dural-based plasmacytoma is a rare pathology that can mimic meningioma, making accurate diagnosis challenging. Although it may be part of systemic MM, it can also present as a solitary lesion with unusual behavior, as demonstrated in our case. Given the lack of specific imaging features, histopathological confirmation and systemic evaluation are crucial. The role of surgery is primarily to aid in diagnosis and relieve symptoms, while chemotherapy and radiotherapy remain the mainstays of treatment when systemic disease is confirmed. Early preoperative workup for MM is vital to guide appropriate treatment, particularly in cases where the pattern of disease is not yet established.
